# Sub-genomic selection patterns as a signature of breeding in the allopolyploid *Brassica napus* genome

**DOI:** 10.1186/1471-2164-15-1170

**Published:** 2014-12-23

**Authors:** Lunwen Qian, Wei Qian, Rod J Snowdon

**Affiliations:** Department of Plant Breeding, IFZ Research Centre for Biosystems, Land Use and Nutrition, Justus Liebig University, Heinrich-Buff-Ring 26-32, 35392 Giessen, Germany; College of Agronomy and Biotechnology, Southwest University, 400716 Chongqing, China

**Keywords:** Rapeseed, Population genomics, LD, Haplotype, Selection

## Abstract

**Background:**

High-density single-nucleotide polymorphism (SNP) genotyping arrays are a powerful tool for genome-wide association studies and can give valuable insight into patterns of population structure and linkage disequilibrium (LD). In this study we used the Brassica 60kSNP Illumina consortium genotyping array to assess the influence of selection and breeding for important agronomic traits on LD and haplotype structure in a diverse panel of 203 Chinese semi-winter rapeseed (*Brassica napus*) breeding lines.

**Results:**

Population structure and principal coordinate analysis, using a subset of the SNPs, revealed diversification into three subpopulations and one mixed population, reflecting targeted introgressions from external gene pools during breeding. Pairwise LD analysis within the A- and C-subgenomes of allopolyploid *B. napus* revealed that mean LD, at a threshold of *r*^2^ = 0.1, decayed on average around ten times more rapidly in the A-subgenome (0.25-0.30 Mb) than in the C-subgenome (2.00-2.50 Mb). A total of 3,097 conserved haplotype blocks were detected over a total length of 182.49 Mb (15.17% of the genome). The mean size of haplotype blocks was considerably longer in the C-subgenome (102.85 Kb) than in the A-subgenome (33.51 Kb), and extremely large conserved haplotype blocks were found on a number of C-genome chromosomes. Comparative sequence analysis revealed conserved blocks containing homoloeogous quantitative trait loci (QTL) for seed erucic acid and glucosinolate content, two key seed quality traits under strong agronomic selection. Interestingly, C-subgenome QTL were associated with considerably greater conservation of LD than their corresponding A-subgenome homoeologues.

**Conclusions:**

The data we present in this paper provide evidence for strong selection of large chromosome regions associated with important rapeseed seed quality traits conferred by C-subgenome QTL. This implies that an increase in genetic diversity and recombination within the C-genome is particularly important for breeding. The resolution of genome-wide association studies is also expected to vary greatly across different genome regions.

**Electronic supplementary material:**

The online version of this article (doi:10.1186/1471-2164-15-1170) contains supplementary material, which is available to authorized users.

## Background

Linkage mapping is a key tool for identifying the genetic basis of quantitative traits in plants. Most agronomic traits in crops are controlled by complex quantitative trait loci (QTL) and their genetic basis is frequently dissected using QTL mapping. In rapeseed (oilseed rape, canola: *Brassica napus* L*.*), the world’s second most important oilseed crop, a vast number of studies have reported QTL for various agronomic, developmental, seed quality and resistance traits since the first genetic mapping of QTL in this species by [1]. Bi-parental genetic mapping populations can be limited by low polymorphism or small population size, however. In addition, only two alleles per locus and few recombination events are considered to estimate the genetic distance between marker loci and to identify the causative genomic regions underlying QTL, thereby limiting the mapping resolution. Although the power of QTL detection in bi-parental mapping populations is generally high, the value of the detected QTL for breeding is often underscored by unpredictable effects in different genetic backgrounds.

Association genetics approaches, utilising genetically unrelated collections or populations of varieties and breeding lines, are a useful alternative for QTL localisation [2]. In contrast to conventional QTL mapping, association mapping is based on linkage disequilibrium (LD). Utilisation of the higher number of historical recombinations in less related populations can greatly improve the mapping resolution compared to a segregating bi-parental population [3]. In recent years association mapping has been broadly adopted for quantitative genetic analyses in crop species [4–6]. A major prerequisite for association mapping is the availability of densely-spaced, molecular markers spanning the entire genome. The discovery and implementation of genome-wide screening for single-nucleotide polymorphism (SNP) markers, even in complex polyploid crop species like *B. napus*, has advanced extremely rapidly in recent years since the introduction of ultrafast DNA sequencing technologies [7]. High-density SNP arrays like the *Brassica* 60 k SNP Illumina consortium array (Illumina, San Diego, CA, USA) have opened the way for high-resolution QTL analyses based on linkage disequilibrium (LD) in both major and minor crops.

A well-known problem with genome-wide association studies (GWAS) is the presence of undetected population structure, which can lead to both false-positive results and a failure to detect genuine associations [8]. Because it also strongly influences LD patterns [2], an accurate estimate and understanding of population structure is critically important for association mapping. On the other hand, LD analyses also provide important insight into the history of both natural and artificial selection (breeding) and can give valuable guidance to breeders seeking to diversify crop gene pools. Recent studies of different *B. napus* ecotypes using collections of genome-wide simple-sequence repeat (SSR) markers gave first insight into genetic diversity and population structure in large collections of *B. napus* [9, 10]. However studies with limited numbers of PCR-based markers are often unable to capture the full extent of LD in diverse populations, and conclusions are limited when data on LD cannot be accurately related to genomic positions of the markers.

The concept of LD describes the non-random association of alleles at two or more loci caused by genetic linkage. Many evolutionary and genetic factors can influence LD. In particular it can reflect the history of natural and artificial selection, mutation, segmental recombination rates, gene conversion and other forces that cause selective sweeps in a genome. Estimates of the extent of LD decay in crop genomes vary depending on the specific species, gene pool or population under investigation. For example, in different sorghum diversity collections a decay of LD was reported to occur within 15–20 Kb [11], 50–100 Kb [12] and 400 kb [13]. Less variation was observed in different populations of maize, with estimates between 0.5-7.0 kb [14–16] and 1–10 kb [17], and rice, with estimates of 20–50 cM [18] and 75–150 Kb [19]. In Arabidopsis LD has been estimated from 50 Kb [20] to over 250 kb [21]. In different *B. napus* populations average LD estimates based on genetic distance measurements were also estimated to vary greatly, from 1–2 cM [22] to more than 20 cM [23].

Some studies have demonstrated that SNPs in strong LD are organised into discrete haplotype blocks that are possibly separated by hotspots of recombination. Genetic variation across the genome is defined by these haplotype blocks, while species-specific block structure is defined by the differential contribution of population history effects in combination with mutation and recombination events. Conservation of haplotype structure may therefore be used for the identification and characterization of functionally important genomic regions during evolution and/or selection. For example, high-resolution analysis of human Y-chromosome haplotypes suggested that a large component of a present-day Asian gene pool originates from Eastern Africa and that Asia was the source of a back-migration to sub-Saharan Africa [24]. Haplotype map analysis in maize found hundreds of selective sweeps and highly differentiated regions that probably contain loci that are keys to geographic adaptation [25]. High-throughput SNP genotyping technologies today enable the use of large numbers of SNPs to construct high resolution LD and haplotype block maps. This is crucial for accurate understanding of associations between markers, genes and phenotypic traits, and at the same time can give more in-depth understanding with regard to species evolution.

Low seed glucosinolate and erucic acid concentrations are two of the most important traits for rapeseed breeding. Both traits have undergone intense purifying selection in elite varieties during the short history of this crop. Detailed analyses of LD and haplotype blocks surrounding major QTL for these two traits [26] will provide valuable new information about selective sweeps and potential linkage drag in the corresponding chromosome areas. At the same time these QTL provide interesting examples to study the dynamics of recent selection signatures at homoeologous trait loci in an important allopolyploid crop species.

*Brassica napus* is a very recent allopolyploid (genome AACC, 2n = 38), derived from only a small number of interspecific hybridisation events between *B. rapa* (AA, 2n = 20) and *B. oleracea* (CC, 2n = 18) within just the past few thousand years [27]. Besides artificially synthesised *B. napus*, only cultivated forms are known, and genetic diversity analyses have revealed only a few eco-geographically and genetically distinct gene pools among cultivated *B. napus* [9, 28]. These suggest that the species may have derived by independent interspecific hybridisation events in Europe and Asia. Today’s Asian semi-winter type rapeseed represents a major intermediate gene pool between European winter-type oilseed rape and spring-sown canola, grown primarily in North America. China’s most important oilseed crop, grown on over 13 million ha, is therefore also a potentially rich source of genetic variation to diversify these narrow gene pools. Chinese rapeseed breeding has extensively used diploid *Brassica* species, particularly *B. rapa*, to enrich the genetic potential of the local gene pool for resistance traits and to improve heterosis. Different *B. napus* gene pools have undergone strict selection for flowering-related traits, including vernalisation requirement, winter survival and photoperiod-dependant flowering, and for essential seed quality traits (primarily low erucic acid and glucosinolate contents). Together with its recent alloploidisation this makes *B. napus* an interesting model for investigating genome-wide and subgenome-specific patterns of genomic and allelic diversification, in the face of broad selective sweeps, during crop domestication.

With these aspects in mind the objectives of this study were: (1) to evaluate genome-specific patterns of population structure and genetic diversity in Chinese semi-winter rapeseed using densely spaced genome-wide SNP markers, (2) to study the extent of LD decay and variation in the distribution of haplotype block size within the A- and C-subgenomes, and (3) to study the effects of intense selection for major seed quality QTL on homoeologous genome regions.

## Results

### Genome-wide SNP polymorphism

From the total of 52,157 SNPs called by the cluster file to be polymorphic in the diversity panel, a stringent BLAST alignment (zero mismatches) of their flanking sequences to the draft *B. napus* reference genome identified 10,065 SNPs with potentially two or more loci in the *B. napus* genome, along with 6,930 SNPs showing no identical BLAST hit. A total of 35,162 single-locus SNPs, each mapping to a single physical genome position, were henceforth implemented for the downstream analyses. For the LD and population structure analysis, 10,168 SNPs with MAF <0.05 were also eliminated, leaving 24,994 high-quality, polymorphic, single-locus SNPs with MAF ≥0.05. The genotype data for these 24,994 SNPs in the diversity panel are provided in Additional file 1 along with their flanking sequence information and expected chromosome positions in the *B. napus* Darmor-*Bzh* reference genome [30].

### Population structure and diversity analysis in the A- and C-subgenomes

**Figure 1:**
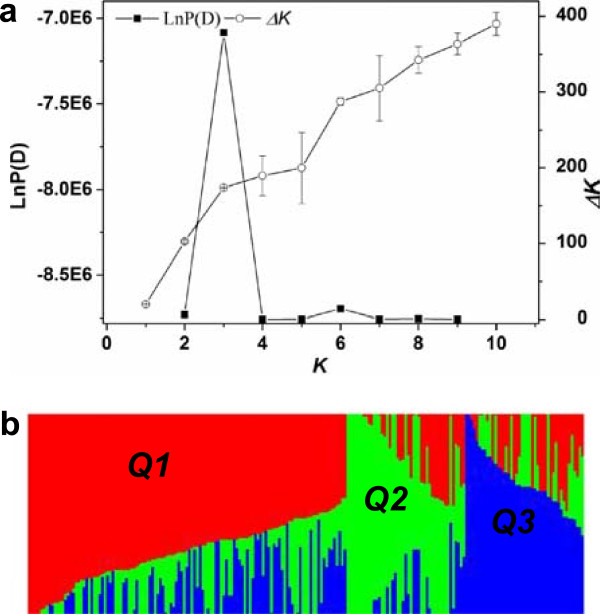
**Analysis of population structure by STRUCTURE in a total of 212** ***Brassica napus*** **accessions genotyped with genome-wide SNP markers. a)** Results of estimated LnP(D) and *∆K* analysis; **b)** Dissection of the genotypes into three distinct subpopulations using *K* = 3.

The results of the population structure analysis measured using the model-based software STRUCTURE are shown in Figure 1a. The LnP(D) value for each given *K* increased together with *K*, the most significant change being observed when *K* increased from 2 to 3. Over all iterations of the *∆K* calculation a much higher likelihood was shown for *K* = 3 than for *K* = 4-10. This suggests the presence of 3 main subpopulations, hereinafter designated Q1, Q2 and Q3 (Figure 1b). Subpopulation Q1 includes 86 Chinese semi-winter, 1 spring and 2 winter rapeseed accessions, while subpopulation Q2 contains 32 Chinese semi-winter rapeseed lines. Q3 contains 27 semi-winter lines, 3 spring-type and 1 winter-type rapeseed. The remaining 60 accessions, including 1 spring-type and 1 winter rapeseed accession, were classified into a mixed subpopulation as they had membership probabilities lower than 0.60 in any given subpopulation (Additional file 2).

**Figure 2:**
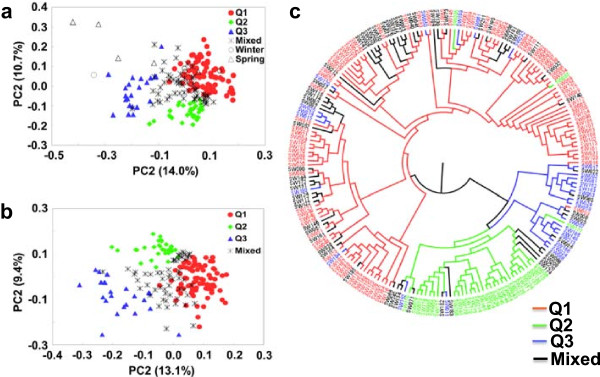
**Principal component analysis (PCA) and unweighted pair group matrix algorithm (UPGMA) tree describing genetic relationships among 212 winter, spring and semi-winter type** ***B. napus*** **accessions.** Q1, Q2 and Q3 are the three subgroups identified by STRUCTURE, assigned with the maximum membership probability, while the mixed subpopulation contains accessions that could not be specifically assigned by STRUCTURE to a single subpopulation. **a)** PCA analysis of 212 semi-winter, winter and spring-type accessions. **b)** PCA analysis of only the 203 Chinese semi-winter accessions. **c)** UPGMA analysis of only the 203 Chinese semi-winter accessions.

The PCA based on Nei’s genetic distance analysis reflected the STRUCTURE results, with the mixed subpopulation clustering in the middle of the three defined subpopulations (Figure 2a). The first principal component (PC1) accounted for 14.0% of the genetic variation and roughly grouped the semi-winter rapeseed into the three main groups Q1, Q2 and Q3. The second principal component (PC2) accounted for 10.7% of the genetic variation and particularly reflected the differentiation between Q1 and Q2.

Comparative analysis of genetic diversity within the three subpopulations revealed higher average PIC and gene diversity in subpopulations Q1 and Q3 than in Q2 (Table 1). The average genetic distance among lines within Q1 (0.38) was very close to that among the winter rapeseed materials (0.36), whereas subpopulation Q3 showed the same average genetic distance as the five spring rapeseed accessions (0.41). Subpopulation Q2 had the lowest average genetic distance of 0.27 (Table 1). Collectively these results suggest introgressions of winter-type oilseed rape genetic background into subpopulation Q1 and spring-type genetic background into Q3, whereas subpopulation Q2 appears to represent a relatively pure genetic background of semi-winter *B. napus*.

PCA and UPGMA tree analysis, using only the 203 Chinese semi-winter genotypes to analyse population structure, resulted in subdivision into the same three subpopulations by PCA analysis, whereby the principal component accounting for genetic diversity was smaller than with inclusion of the outliers in the full set of 212 lines (Figure 2a and b). The results of the UPGMA tree analysis corresponded with around 91% similarity to the PCA (Figure 2c).

**Table 1:** **Summary statistics for genetic diversity within a subset of 154** ***Brassica napus*** **accessions, representing the semi-winter type oilseed rape subpopulations Q1, Q2 and Q3 along with spring (5 accessions) and winter rapeseed (4 accessions)**

Type	Subpopulation	No. of accessions	Genetic distance	Gene diversity	PIC
Semi-winter	Q1	86	0.38	0.34	0.27
	Q2	32	0.27	0.25	0.20
	Q3	27	0.41	0.34	0.27
Spring		5	0.41		
Winter		4	0.36		

**Figure 3:**
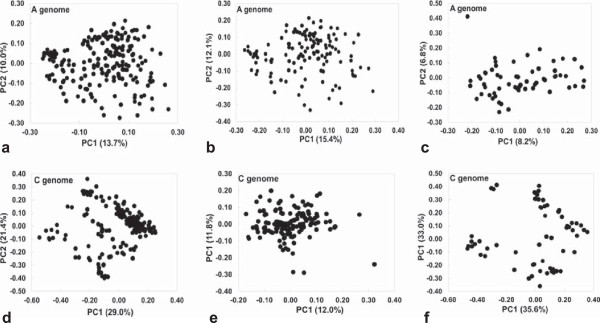
**Comparative principal coordinate analysis (PCA) of subgenomic genetic diversity, measured across 10,750 SNP markers per** ***B. napus*** **subgenome. a,b,c)** A-subgenome diversity; **d,e,f)** C-subgenome diversity; **a,d)** Analysis using all 203 semi-winter accessions**; b,e)** Analysis using 136 accessions with maximal A-subgenome diversity; **c,f)** Analysis using 67 accessions with maximal C-subgenome diversity.

Detailed comparisons of population structure and genetic diversity in the A- and C-subgenomes, estimated using 10,750 randomly selected, unique SNPs with MAF ≥ 0.05, are shown in Figure 3 and Table 2. In the A-subgenome, the first and second principle components explained 13.7 and 10.0% of the genetic diversity. In the C-subgenome, the genetic diversity explained by the first and second principle components was more than double that in the A-subgenome, comprising 29.0 and 21.4%, respectively. However, gene diversity and PIC were higher in the A-subgenome (0.373 and 0.298, respectively) than in the C-subgenome (0.339 and 0.276, respectively; Table 2), suggesting that a small number of accessions had particularly high allelic diversity in some C-subgenome chromosome regions. Hence, C-subgenome SNPs contributing to extreme PCA values were used to further subdivide the subpopulations based on allelic diversity.

**Table 2:** **Comparative analysis of genetic diversity in the A- and C-subgenomes within subsets of Chinese semi-winter rapeseed inbred lines representing the total diversity (203 accessions), maximal A-subgenome diversity (135 accessions) and maximal C-subgenome diversity (68 accession), respectively**

Genome	Subset of 203 accessions		Subset of 135 accessions		Subset of 68 accessions	
	Gene diversity	PIC	Gene diversity	PIC	Gene diversity	PIC
A	0.373	0.298	0.365	0.292	0.377	0.300
C	0.339	0.276	0.277	0.225	0.392	0.310

PIC: Polymorphism information content.

In 135 of these accessions, the first and second principle component analysis accounted for 15.4 and 12.1% genetic diversity in the A-subgenome, compared to only 12.0 and 11.8% in the C-subgenome. Among these materials the gene diversity (0.365) and PIC (0.292) were also higher in the A-subgenome than the C-subgenome (0.277 and 0.225, respectively) (Table 2). In another group of 68 accessions, on the other hand, the first and second principle components explained only 8.2 and 6.8% when A-subgenome SNPs were used, but 35.6 and 33.0%, respectively, with C-subgenome SNPs. Accordingly, in these 67 accessions the gene diversity (0.377) and PIC (0.300) were also lower in the A-subgenome than the C-subgenome (0.392 and 0.310, respectively) (Table 2). Collectively these results suggest that the A-subgenome contributes more genetic diversity to Chinese semi-winter rapeseed than the C-subgenome, but also that a small group of materials appears to have benefited from targeted introgressions of C-subgenome diversity.

### Relative kinship

**Figure 4:**
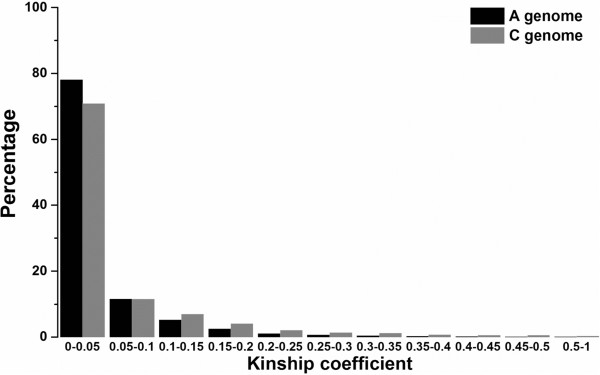
**Comparative analysis of kinship coefficients in the A-subgenome (black bars) and C-subgenome (grey bars) among 203 Chinese semi-winter rapeseed accessions.**

Analysis of kinship using 4000 unique SNPs each from the A- and C-subgenomes, all with MAF ≥ 0.05, supported the finding that the A-subgenome carries more overall genetic diversity than the C-subgenome (Figure 4). At the same time the kinship analysis showed only weak or no relationship among the materials. This might be attributed to the introgression of different rapeseed ecotypes and closely related species.

### Patterns of LD across the semi-winter rapeseed genome

**Figure 5:**
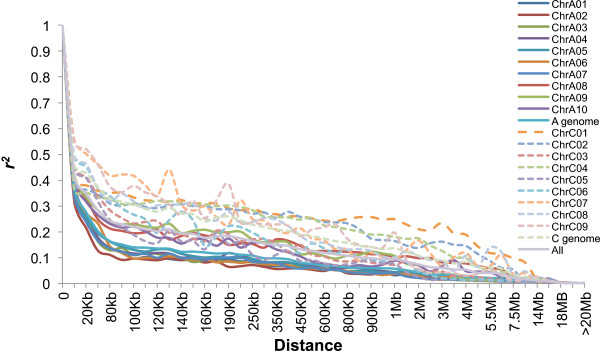
**Patterns of linkage disequilibrium (LD,** ***r*** ^***2***^ **= 0.1) across the 19 haploid chromosomes of semi-winter type** ***B. napus*** **, measured with 24,994 single-copy SNP markers.** The solid lines represent LD decay in A-subgenome chromosomes, while the dashed lines represent LD decay in C-subgenome chromosomes.

To assess patterns of LD extent in more detail, we measured the physical distance at which the pair-wise genotypic association in the filtered SNP dataset decays below a threshold of *r*^*2*^ = 0.1. This revealed huge differences in LD decay between different chromosomes, with LD extending from 0.08-0.09 Mb (chromosome A02) up to 7.00-7.50 Mb (C01, C07 and C08) (Table 3). Figure 5 and Table 3 compare the distribution of *r*^*2*^ with respect to the physical distance over the 19 chromosomes, as well as overall across each subgenome. Considerably faster mean LD decay was observed on A-subgenome chromosomes (0.25-0.30 Mb) than C-subgenome chromosomes (2.00-2.50 Mb; Table 3).

**Table 3:** **Average distance of linkage disequilibium (LD) decay (** ***r*** ^***2***^ **= 0.1) on A- and C-subgenome chromosomes, calculated using 24,994 unique, genome-wide SNP markers with minor allele frequency (MAF) ≥0.5, in a collection of 203 Chinese semi-winter** ***B. napus*** **accessions**

Subgenome	Chromosome	LD decay (Mb)	No. of SNPs
A-subgenome	A01	0.11-0.12	1117
	A02	0.08-0.09	891
	A03	0.14-0.15	1646
	A04	0.20-0.25	1139
	A05	0.18-0.19	1249
	A06	0.13-0.14	1153
	A07	0.13-0.14	1412
	A08	1.50-2.00	820
	A09	1.00-1.50	1166
	A10	0.45-0.50	1171
	Mean	0.25-0.30	1176
C-subgenome	C01	7.00-7.50	2041
	C02	5.00-5.50	1891
	C03	0.60-0.65	2094
	C04	3.50-4.00	2473
	C05	0.40-0.45	718
	C06	0.80-0.85	905
	C07	7.00-7.50	1285
	C08	7.00-7.50	1156
	C09	1.00-1.50	667
	Mean	2.00-2.50	1581
A + C	Mean	0.85-0.90	1378

### Subgenome-specific haplotype block structure

The same markers used for LD estimation were employed to estimate haplotype blocks in the 203 semi-winter rapeseed accessions. In the A-subgenome, the mean MAF per chromosome varied from 0.20 (A08) to 0.31 (A09, A10), with a mean of 0.27 over all A-subgenome chromosomes. The mean *r*^*2*^ per A-subgenome chromosome varied from 0.17 (A02, A03) to 0.36 (A09) with a mean of 0.23 over the whole A-subgenome (Table 4). On C-subgenome chromosomes mean MAF varied from 0.19 (C02) to 0.29 (C04, C05), with an average of 0.24 over the whole C-subgenome. The mean *r*^*2*^ on C-subgenome chromosomes was considerably higher, ranging from 0.41 (C05, C07) to 0.78 (C04) with an average of 0.59 over the whole C-subgenome (Table 4). The higher MAF in the A-subgenome and stronger LD in the C-subgenome further indicate a higher genetic diversity of the A-subgenome than the C-subgenome.

**Table 4:** **Chromosome-specific haplotype block structure analysed using in a collection of 203 Chinese semi-winter** ***B. napus*** **accessions**

Chromosome	No.of SNPs	Chromosome length (Mb)	Mean MAF	Mean ***r*** ^***2***^	Number of blocks	Mean block size (Kb)	Block coverage area per chromosome (Mb)	Block coverage percentage per chromosome (%)
A01	1117	23	0.27	0.19	190	27.74	5.27	0.23
A02	891	25	0.25	0.17	145	23.30	3.38	0.14
A03	1646	29	0.27	0.17	286	21.84	6.25	0.22
A04	1139	20	0.24	0.19	175	26.46	4.63	0.23
A05	1249	23	0.27	0.21	215	33.07	7.11	0.31
A06	1153	24	0.28	0.20	213	32.07	6.83	0.28
A07	1412	24	0.28	0.19	247	24.57	6.07	0.25
A08	820	19	0.20	0.33	129	51.72	6.67	0.35
A09	1166	33	0.31	0.36	157	60.57	9.51	0.29
A10	1171	17	0.31	0.30	167	33.78	5.64	0.33
A-subgenome mean	1176	24	0.27	0.23	192	33.51	6.14	0.26
C01	2041	39	0.20	0.73	135	119.92	16.19	0.42
C02	1891	46	0.19	0.84	124	186.10	23.07	0.50
C03	2094	60	0.23	0.46	209	89.810	18.77	0.31
C04	2473	49	0.29	0.78	172	100.58	17.30	0.35
C05	718	43	0.29	0.41	92	50.64	4.66	0.11
C06	905	37	0.27	0.46	113	82.04	9.27	0.25
C07	1285	45	0.25	0.41	138	95.46	13.17	0.29
C08	1156	38	0.21	0.61	124	93.45	11.59	0.31
C09	667	48	0.23	0.60	66	107.67	7.11	0.15
C-subgenome mean	1581	33	0.24	0.59	130	102.85	13.46	0.41
Whole genome mean	1378	29	0.25	0.41	161	68.18	9.80	0.34

MAF: Minor allele frequency.

**Figure 6:**
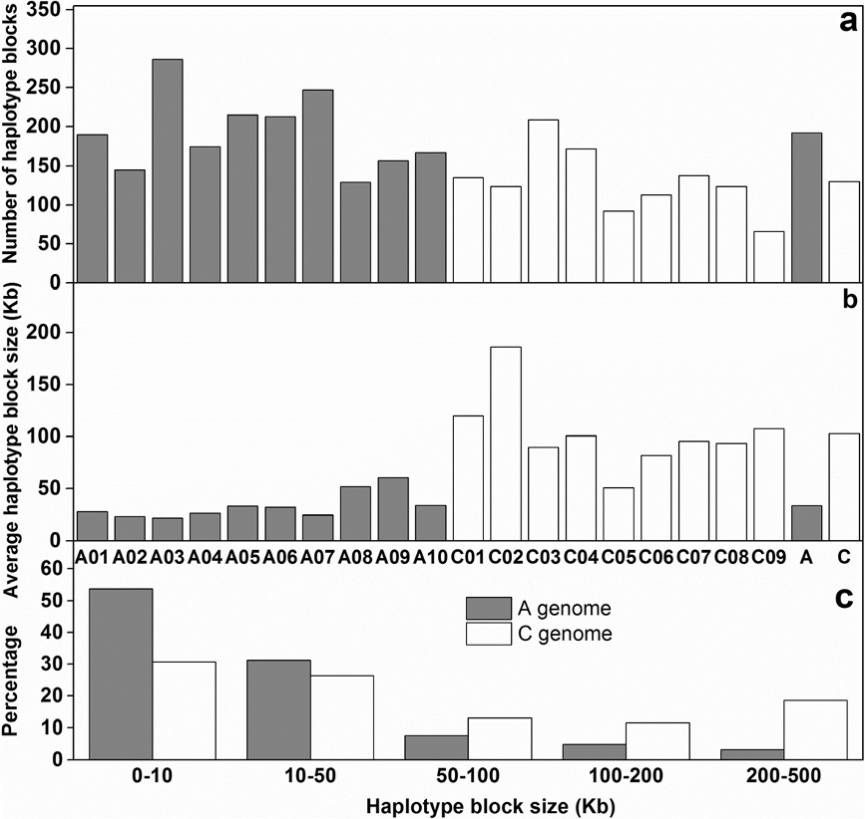
**Comparative analysis of haplotype block structure in the A-subgenome (grey bars) and C-subgenome (black bars) of semiwinter-type** ***Brassica napus.*** **a)** Comparison of the numbers of LD blocks on A- and C-subgenome chromosomes. **b)** Comparison of the average size of LD blocks on A- and C-subgenome chromosomes. **c)** Comparison of the size range distributions of haplotype blocks in the A- and C-subgenomes.

A summary of the distribution, size and number of haplotype blocks per chromosome is presented in Table 4. A total of 3,097 conserved haplotype blocks were detected in the 203 Chinese semi-winter rapeseed accessions, spanning 182.49 Mb (15.17% of the assembled reference genome). In the A-subgenome chromosomes, mean haplotype block number ranged from 129 (A08) to 286 (A03) with an average of 192, while the mean haplotype size ranged from 21.84 (A03) to 60.57 Kb (A09) with an average of 33.51 Kb. The mean haplotype block number in C-subgenome chromosomes varied from 66 (C09) to 209 Kb (C03) with an average of 130 Kb, while mean haplotype size was considerably larger, ranging from 50.64 (C05) to 186.10 Kb (C02) with an average of 102.85 Kb (Table 4; Figure 6a and b). In the A-subgenome 53.85% and 30.72% of haplotype blocks ranged in size from 0–10 Kb and 10–50 Kb, respectively, whereas only 31.20% of C-subgenome haplotype blocks were in the 0–10 Kb size range and only 26.34% in the 10–50 Kb size range (Figure 6c). In contrast, much fewer regions with long-range haplotype conservation were observed in the A-subgenome, where haplotype blocks ranging in size from 50–100 Kb, 100–200 Kb and 200–500 Kb were present at frequencies of only 7.69, 4.89 and 3.27%, respectively. In the C-subgenome, on the one hand, the respective haplotype block sizes were observed at much higher frequencies of 13.04, 11.60 and 18.58%, respectively (Figure 6c), demonstrating that the higher mean haplotype block size in the C-subgenome is caused by retention of long-range LD.

Particularly high conservation of LD was observed on chromosomes C01, C02, C04 and C09, which showed very high average *r*^*2*^ values of 0.73, 0.84, 0.78 and 0.60, respectively, and correspondingly large mean haplotype block sizes of 119.92, 186.10, 100.58 and 107.67 Kb, respectively (Table 4).

### Analysis of homoeologous QTL regions

Comparative sequence analysis revealed conserved haplotype blocks and LD corresponding to homoeologous QTL for seed glucosinolate content on homoeologous chromosomes A02/C02 and A09/C09, respectively, and for erucic acid content on chromosomes A08 and C03. The genomic positions of known QTL for seed glucosinolate content on chromosome A02/C02 (mapped in detail by [26]) were localised by a BioEdit local BLAST search [41] using 17 and 5 SNPs, respectively, within the QTL confidence intervals. These SNPs mapped to overlapping homoeologous regions from 19,680,403 – 23,996,416 bp on chromosome A02 and from 41,859,157 bp – 44,499,708 bp on chromosome C02, respectively (Additional file 3). Similarly, 17 SNPs spanning another major QTL for seed glucosinolate content on chromosome A09 [26] were localised to overlapping homoeologous regions from 775,293 – 3,831,394 bp and 290,810 – 5,109,219 bp on chromosomes A09 and C09, respectively (Additional file 3). Insufficient SNPs were present in the QTL on chromosome C09 from [26], hence the physical region on C09 was predicted by a BLAST search of SNPs from the homoeologous QTL region on A09.

**Figure 7:**
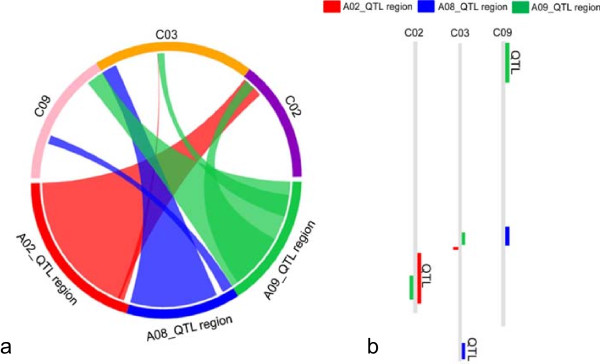
**Synteny alignments of QTL for seed glucosinolate (chromosomes A02 and A09) and erucic acid (A08) into homoeologous regions of chromosomes C02, C09 and C03, respectively. a)** Circular map and **b)** linear map, respectively, showing showing A-subgenome chromosomal positions corresponding to C-subgenome QTL.

Homoeologous QTL for erucic acid content on chromosomes A08 and C03 [26] were physically localised using 17 and 5 SNPs, respectively, spanning these two loci. The corresponding QTL covered the regions from 9,513,648 – 12,196,483 bp and 54,259,136 – 57,154,658 bp on chromosomes A08 and C03, respectively (Additional file 3). As expected, these regions include the two *B. napus* homologues of the gene *FATTY ACID ELONGASE 1* (*Bna.FAE1*) that carry the agronomically essential low erucic acid mutations [44]. Results from matching of the physical positions were compared to BLAST alignments of sequences against each other with similar results (Additional file 3; Additional file 4; Figure 7).

### Comparative analysis of haplotype blocks within homoeologous QTL for key traits

**Figure 8:**
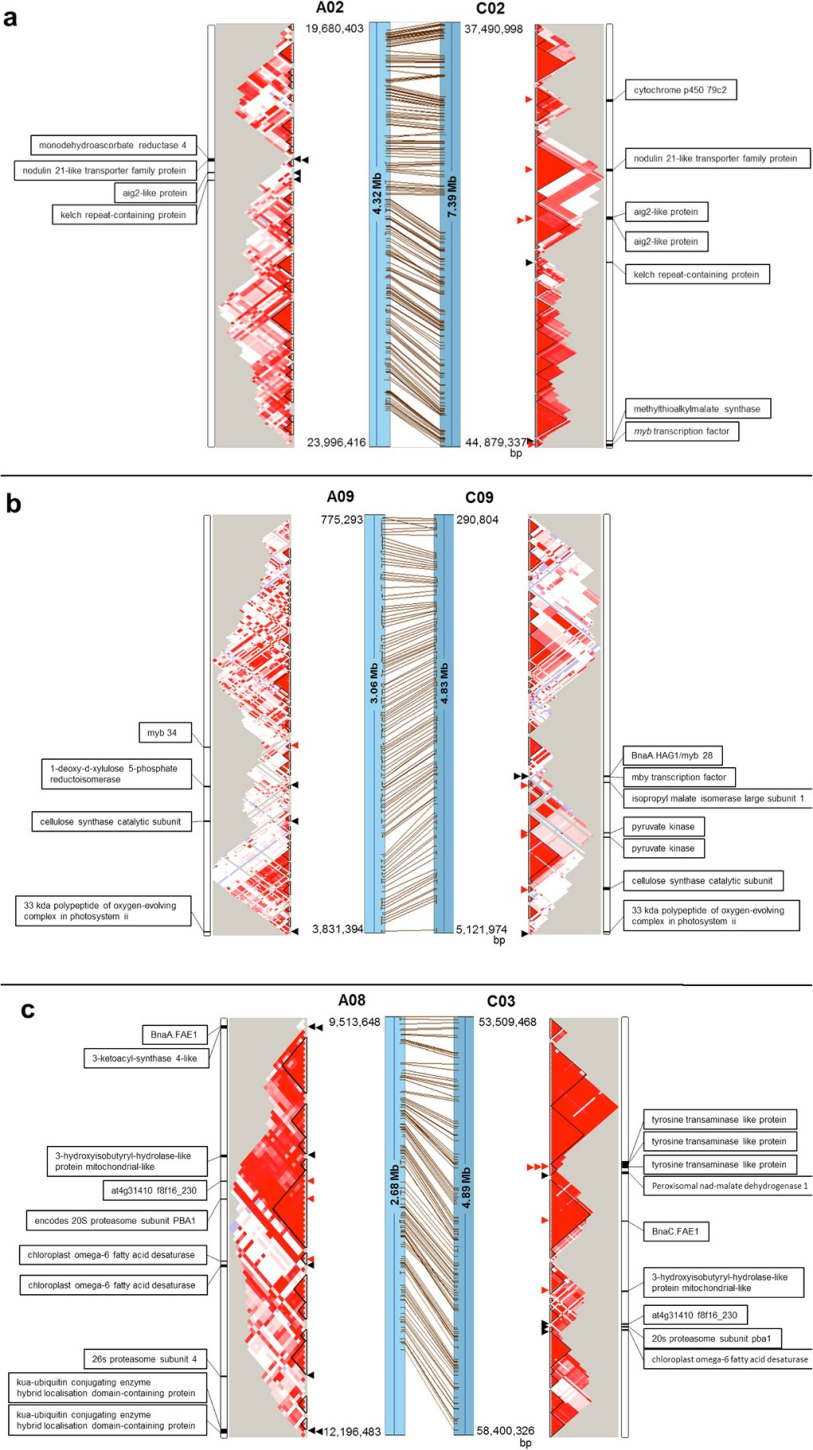
**Comparative sequence analysis showing differences in linkage disequilibrium (LD) and putative functional candidate gene content within haplotype blocks between homoeologous A-subgenome and C-subgenome QTL for (a,b) seed glucosinolate (GLS) and (c) erucic acid content on chromosomes (a) A02/C02, (b) A09/C09 and (c) A08/C03, respectively.** Regions with strong (D’ = 1), high confidence LD (LOD > 2) are plotted bright red, lighter shades of red represent moderate LD (D’ < 1) with high confidence (LOD > 2), while light blue blocks represent weak, low confidence LD (D’ = 1, LOD < 2) and white blocks an absence of LD (D’ < 1, LOD < 2). Connecting lines show syntenic sequence alignments between the homoeologous QTL regions. Arrows show putative function candidate genes annotated to **(a,b)** glucosinolate biosynthesis or catalysis or **(c)** fatty acid or oil biosynthesis. Red arrows indicate genes under strong selection within conserved LD/haplotype blocks, whereas black arrows indicate that the corresponding gene at the homoeologous locus is not within a regions under strong selection. Gene names are given opposite the arrow positions on the homoeologue on which they are present.

Estimates of *D'* based on marker pairs lying within homoeologous QTL for seed glucosinolate (GLS*,* chromosomes A02/C02 and A09/C09) and erucic acid content (A08/C03) revealed large differences in recombination structure and extent of LD between the respective homoeologous chromosome regions (Figure 8). Two QTL for GLS described by Delourme *et al*. [26] were physically mapped to 4.32 and 3.06 Mb regions of chromosomes A02 and A09, whereas the corresponding homoeologous regions on chromosome C02 and C09 covered 7.39 and 4.83 Mb, respectively. On A02 and A09 the mean *r*^*2*^ (0.23 and 0.12) and mean haplotype block sizes (20.67 and 12.46Kb) are both considerably smaller than in the homoeologous regions on C02 and C09 (0.45 and 0.21, 120.35 and 26.27 Kb, respectively) (Table 5; Figure 8). A similar observation was made for the eurcic acid content QTL region described by Delourme *et al.* [26], which mapped to a physical region covering 2.68 Mb of chromosome A08 and a homoeologous region of 4.89 Mb on chromosome C03. On A08 the erucic acid QTL region shows considerably lower LD (mean *r*^*2*^ = 0.35) and mean haplotype block size (56.17 Kb) than the homoeologous QTL region on C03 (mean *r*^*2*^ = 0.45, mean haplotype block size 181.29 Kb) (Table 5; Figure 8).

**Table 5:** **Comparative sequence analysis among haplotype blocks showing conserved linkage disequilibrium (LD) covering homoeologous quantitative trait loci (QTL) for seed glucosinolate (GSL; chromosomes A02/C02 and A09/C09, respectively) and erucic acid content (chromosomes A08 and C03)**

Chromosome	GLS QTL region (bp)	Erucic acid QTL region (bp)	Region size (Mb)	No. of blocks	Mean block size (Kb)	LD block coverage (Mb)	Mean ***r*** ^***2***^
A02	19,680,403-23,996,416		4.32	24	20.67	0.49	0.23
C02	37,490,998-44, 879,337		7.39	31	120.35	3.73	0.45
A09	775,293-3,831,394		3.06	37	12.46	0.46	0.12
C09	290,804-5,121,974		4.83	26	26.27	0.68	0.21
A08		9,513,648-12,196,483	2.68	12	56.17	0.67	0.35
C03		53,509,468-58,400,326	4.89	17	181.29	3.08	0.45

We further analyzed these homoeologous QTL regions for genes related to seed GLS and erucic acid content, respectively. Three GSL biosynthetic process genes and one GSL catabolic process gene were located in a 0.50 Mb region with disrupted LD that spanned the QTL for GSL on A02 (Additional file 5; Figure 8). In contrast the homoeologous QTL region on chromosome C02 contained seven GSL-related genes (including the *myb* transcription factor) that spanned a 5.7 Mb region with extensive LD. This region included four glucosinolate biosynthetic process genes, within three conserved haplotype blocks ranging in size from 38 to 326 Kb (Additional file 5; Figure 8). Similar patterns of LD conservation were observed between the homoeologous QTL for GSL on chromosomes A09 and C09. On A09, four GSL biosynthetic process genes (including a *myb34*) were located within a 1.12 Mb region with low LD. In contrast, seven genes implicated in GSL biosynthesis (including a *myb* transcription factor and the important GSL gene *BnaA.HAG1*/*myb28* [67]) were located in a 1.98 Mb region, including four GSL biosynthesis genes, within three conserved haplotype blocks ranging in size from 21 to 133 Kb (Additional file 5; Figure 8).

On chromosome A08, the major QTL for erucic acid content was found to contain five additional fatty acid biosynthetic process genes besides the causal gene *BnaA.FAE1*. These included a *3-ketoacyl-synthase 4-like* gene and two *chloroplast omega-6 fatty acid desaturase* orthologues, along with four fatty acid beta-oxidation genes. Within a total length of 1.93 Mb we found that two fatty acid beta-oxidation genes and one fatty acid biosynthetic process gene (*chloroplast omega-6 fatty acid desaturase*) were located in separate haplotype blocks, ranging in size from 13 to 137 Kb (Additional file 5; Figure 8). Within the corresponding homoeologous QTL region on chromosome C03 we localised six fatty acid biosynthetic process genes (including *BnaC.FAE1* and a *chloroplast omega-6 fatty acid desaturase*) and three fatty acid beta-oxidation genes, covering a total of 2.54 Mb. Four of the fatty acid biosynthesis genes (including *BnaC.FAE1*) and one fatty acid beta-oxidation gene were located in strongly conserved haplotype blocks ranging in size from 281 to 477 Kb (Additional file 5; Figure 8).

The different intensities of selection at A- and C-subgenome QTL for seed erucic acid and GSL content were confirmed by reanalyzing the extent of LD conservation based on *r*^2^ with LDheatmap (Additional file 6). Again we found strong LD conservation and similar gene content within the C-subgenome QTL, whereas A-subgenome QTL showed less conserved LD and more fragmented haplotype structure (Additional file 5, Additional file 6). The results suggest considerably stronger retention of C-subgenome haplotype blocks than A-subgenome haplotypes within these important seed quality QTL.

## Discussion

### Population structure and genetic diversity

Rapeseed breeding materials in Australia and China have similar origins, with introductions from Europe, Canada and Japan in the mid-20th century and subsequent interchange of germplasm since that time [45]. Recently, hybrid breeding has received considerable attention, with development of genetically diverse gene pools through recurrent, reciprocal selection of genetic diversity from different *B. napus* ecotypes [9]. The three main population subgroups we observed in our diversity panel may reflect breeding efforts to diversify Chinese semi-winter rapeseed by introgressing genetically distant winter rapeseed (in the case of Q1) and spring canola (in the case of Q3) into different hybrid breeding pools.

Genetic diversity in Chinese *B. napus* has been further improved by introgressions from Asian *B. rapa* [45, 46], the diploid donor of the *B. napus* A-subgenome. According to Liu [47] and Shiga [48], more than 50% of *B. napus* cultivars in China and Japan are derived from *B. napus* × *B. rapa* crossings. Correspondingly, we observed considerably more genetic diversity in the A-subgenome of Chinese rapeseed than the C-subgenome. interestingly, however, we found 67 accessions with a stronger diversity in the C-subgenome than the A-subgenome. These may derive from programs to introgress additional diversity and resistance alleles from C-genome donors.

### LD and haplotype block analysis

Around 15.17% of the assembled *B. napus* genome could be assigned to haplotype blocks, with large gaps between blocks (data not shown). With an average SNP density of only one SNP per 48.01 Kb, it is difficult to detect very small haplotype blocks [49]. Recent studies in maize [25] and Arabidopsis, [50] have demonstrated the power of comprehensive genome-wide SNP genotyping arrays for generation of detailed haplotype maps and high-resolution LD analysis. Whole-genome resequencing data provides the ultimate dimension to uncover LD in association with signatures of natural and artificial selection, but so far has been limited to species with relative small, diploid genomes, like sorghum [51]. Many of the problems with duplicated SNP loci on the Brassica 60 k SNP array result from the extensive recent genome duplications which make it quite challenging to design locus-specific SNP assays in many strongly homoeologous regions of the genome. These technical difficulties can cause a reduction in resolution in some genome regions. Nevertheless, use of a high-density SNP array to analyse and compare LD and selection in homoeologous QTL is a unique feature of this study in comparison to previous work in simple diploid species. As a recent allopolyploid *B. napus* thus provides interesting insight into the evolutionary processes of selection in an important crop [30].

The 24,994 unique, polymorphic SNPs we used in our analyses were nevertheless sufficient to perform a preliminary whole-genome analysis of haplotype block structure in *B. napus*. In particular we were able to demonstrate that some *B. napus* chromosomes carry extremely large segments of highly conserved LD, and that this phenomenon is a particular feature of C-subgenome chromosomes. This may indicate increased recombination rates of A-subgenome chromosomes after interspecific hybridisations with *B. rapa*. Boosts of homologous recombination among diploid chromosome pairs after interspecific hybridisation were documented in *Brassica* crosses by Leflon *et al*. [52]; this might have caused more rapid LD decay and subsequently shorter-range haplotype blocks in A-subgenome chromosomes in the present materials after hybridisations with *B. rapa*. Although breeders have used interspecific crosses to improve agronomic traits and increase C-subgenome genetic diversity in *B. napus*, it is extremely difficult to obtain viable hybrid seeds from *B. napus* × *B. oleracea* crosses [53, 54], causing a constraint in the ability to diversify the C-subgenome genetic component. It is thought that *B. napus* arose only in post-neolithic times and from only a small number of independent hybridisation events [27], and that the Chinese rapeseed genepool may predominantly represent only one or a few of these events. Hence it is perhaps not surprising that recombination and diversity appear to be considerably lower in the C-subgenome of Chinese oilseed rape. An alternative explanation, which may also partly explain the great overall difference in LD between the A and C subgenomes, is the considerably greater expansion of transposable elements in the *B. napus* C-subgenome compared to the A-subgenome [30], since transposon-rich regions are often observed to be recombination-poor [55]. On the other hand, this fails to explain the great variation in the size of LD and long-range haplotype blocks we observed among different C-subgenome chromosomes. A more simple contributing factor is likely to be strong natural and artificial selection for key adaptation and seed quality traits, where specific variants seem to have been selected during the face of ecogeographical adaptation and human selection, for example for flowering time or quality traits. Strong selection at a locus is expected to reduce diversity and increase LD and haplotype block size in the surrounding region [56].

In particular, stronger LD and longer-range LD blocks on chromosomes C01, C02, C04 and C09 suggest particularly strong selection the corresponding region of these chromosomes. According to Liu [47] rapeseed was introduced into China from Europe in the 1930-1940s, although a later origin within the past few hundred years in Japan is also postulated [57, 58]. Guryev *et al*. [59] showed that the evolutionary selection process drives conservation of long-range allele combinations, causing chromosome regions to retain a long-range haplotype block structure. Artificial selection can also have a profound effect on LD in crop plants, with selection for key agronomic traits like flowering behavior, resistances or essential quality parameters causing genetic bottlenecks that lead to extensive conserved haplotype blocks in chromosome regions carrying the responsible gene loci or major QTL for selected traits. Modern double-low quality oilseed rape has undergone selective sweeps for reduction of seed erucic acid and glucosinolate contents, along with flowering time, winter hardiness and vernalisation-related traits. Such selection tends to reduce allele diversity and increase haplotype block structure around the major responsible loci, however detailed studies of LD conservation in oilseed rape breeding pools on a DNA sequence level has only recently become possible since the availability of high-density genome-wide SNP markers [7] in combination with annotated *Brassica* genome sequences. Here we identified chromosome-scale LD patterns in *B. napus* genome regions carrying important QTL for both a simple, bigenically inherited trait (erucic acid content) and for a complex quantitative trait (glucosinolate content).

The observed distance of LD decay in Chinese oilseed rape was 0.85-0.90 Mb, which is higher than maize with 0.5-10 kb [17] and Arabidopsis with 50–250 Kb [21]. This reflects the very recent domestication of *B. napus*, its exclusive use in cultivation, with no known wild forms, and the strong selection bottlenecks associated with cultivation and breeding. Previous studies (e.g. Wang et al. [60]) have shown that the A-subgenome has been successfully improved by closely related species, leading to more rapid decay of LD in the A-subgenome than the C-subgenome. Our results showed that the relatively low overall LD conservation in Chinese rapeseed is caused mainly by a lack of genetic diversity in the C-subgenome. According to Mei *et al*. [61], natural *B. napus* has very low genetic diversity compared with its diploid progenitors, therefore intercrossing with the parental species can be an effective way to broaden genetic diversity in rapeseed. To achieve this it may be necessary to overcome sexual compatibility barriers by using embryo rescue techniques, for example. In recent years considerable progress has been made in introducing novel C-genome donors to European winter oilseed rape, in order to improve genetic diversity particularly for disease resistance [62–64] or heterosis [65].

### Haplotype block and extent of LD of homologues QTL region

Conserved haplotype blocks with strong LD spanning major homoeologous QTL for seed GLS (chromosomes A02/C02 and A09/C09) and erucic acid (A08/C03) reflect the strong selection bottlenecks for these traits. On the other hand, the introgression of exotic A-subgenome diversity from *B. rapa* has apparently led to shorter-range haplotype blocks and lower LD in A-subgenome than C-subgenome QTL. Chinese *B. napus* originated from Europe [47], being introduced to China in the 1930-1940s and replacing the traditional oilseed crop *B. rapa*. Local adaptation to the new ecogeographical environment, and diversification of breeding pools, was achieved by introgressing local populations of the wild progenitors and closely related species, particularly *B. rapa* [66–68]. Our results show that this process resulted in substantial decay of LD surrounding important A-subgenome QTL, whereas longer-range haplotype blocks and higher LD are retained around C-subgenome QTL regions. Importantly, conserved haplotype blocks in C-subgenome QTL tend to retain multiple genes related to relevant biosynthetic processes, which can potentially cause linkage drag that slows breeding progress for the trait of interest.

Various forces have potentially contributed to haplotype conservation in C-subgenome QTL in *B. napus*, including genetic bottlenecks from artificial or natural selection or a simple lack of recombination and sequence diversity. We found rates of sequence polymorphism to be generally lower in C-subgeneome QTL regions than their corresponding A-subgenome homoeologues, suggesting that the former may be the dominant mechanism. On the other hand a suppression of recombination, due to the increased density of transposable elements in the C-subgenome [30], cannot be ruled out. Detailed haplotype block analysis of important QTL can help in the precise mapping of important genomic regions and location of favorable alleles. In association with genomic sequence data it can also help to more precisely predict quantitative trait-related genes (QTG) in QTL regions using targeted association mapping with high-density markers.

The strongly conserved LD we observed across the QTL on chromosomes C02 and C09 was found to be associated with a large number of functionally related genes in close genetic linkage. The corresponding homoeologous QTL on chromosomes A02 and A09 each contained fewer genes annotated to the QTL function. This result demonstrates the important role of gene loss during or after allopolyploidisation in natural and/or artificial selection of key traits like GSL content [30]. Natural evolution results in a positive and balancing selection within the genome, whereas artificial secletion can lead to partial separation of phenotypic traits. According to Harper *et al*. [69], deletions affecting homologues of the GSL biosynthesis gene *Bna.HAG1*/*myb28* resulted in selective sweeps affecting the QTL for GSL on A09 and C02. Both in this case, and in the case of homologous QTL erucic acid content on chromosomes A08 and C03, we demonstrate that selective sweeps can also incorporate additional, functionally-related genes for which alleles in strong LD may have either a positive or negative influence (linkage drag) on target traits (e.g. GSL content, fatty acid composition or oil content). Detailed analysis of LD structure and signatures of selection in important QTL can guide breeders towards a knowledge-based crop improvement by genome-based introgression of useful genetic diversity.

## Conclusions

Using densely-spaced genome-wide SNPs to analyse subgenomic genetic diversity in semi-winter *B. napus*, we found stronger LD and long-range haplotype conservation in C-subgenome chromosomes. Comparative sequence analysis revealed conserved blocks containing homoloeogous QTL for important seed quality traits under intense artificial selection. The results indicate strong selection for large chromosome regions associated with important seed quality traits conferred by C-subgenome QTL, suggesting that an increase in genetic diversity and recombination within the C-genome is particularly important for breeding. The resolution of genome-wide association studies is also expected to vary greatly across different genome regions.

## Methods

### Plant germplasm and genotyping

A set of 203 homozygous *B. napus* inbred lines was collected to construct a diversity panel broadly representing variability in Chinese semi-winter rapeseed. The materials (Additional file 2) were obtained as self-pollinated seeds from Southwest University, Chongqing, China, where they represent part of a breeding program spanning genetic diversity from the broader Asian gene pool. In addition, five spring-type and four winter-type *B. napus* inbred lines were included as outliers to assess the extent and impact of introgressions from extant gene pools into the Asian semi-winter materials.

DNA was extracted by a modified CTAB procedure according to Murray and Thompson [29]. The Brassica SNP consortium 60 k Infinium genotyping array (Illumina Inc., San Diego, CA, USA) was used to obtain high-density genome-wide data from each accession, according to the manufacturer’s protocol. DNA samples were analysed by a commercial genotyping service company (TraitGenetics, Gatersleben, Germany) and SNP calling was performed using a proprietary cluster file generated by the International Brassica SNP consortium which designed the array (Isobel Parkin, AAFC, Saskatoon, SK, Canada, personal communication). A pre-publication draft assembly of the *B. napus* ‘Darmor-Bzh’ reference genome assembly [30] was kindly provided by Boulos Chalhoub (INRA-UNRV, Évry, France) for assignment of physical genome positions of the SNPs.

### Genetic diversity and population structure analysis

Analyses of gene diversity, polymorphic information content (PIC) and genetic distance [31] were performed using the software PowerMarker version 3.25 [32]. The population structure among the 212 accessions in the panel was assessed using the model-based Bayesian clustering method implemented in STRUCTURE version 2.3.3 [33]. The number of subgroups (*K*) was set from 1 to 10. For each *K*, seven runs were performed separately with burn-in length and iterations set to 10000 and 50000, respectively. Lines with membership probabilities *≥*0.6 were assigned to the corresponding subgroups and lines with membership probabilities <0.6 were assigned to a “mixed” subgroup.

The software Powermarker version 3.25 [32] was employed to calculate genetic distance among accessions according to Nei [31]. The double-centred genetic matrices thus created were used to obtain eigenvectors by implementing the modules DCENTER and EIGEN in the software NTSYSpc 2.1 [34]. In combination with the population structure result from STRUCTURE, the first and second principle components from these data were used to prepare 2D plots using Microsoft Office Excel 2010. An unweighted pair group matrix algorithm (UPGMA) tree was calculated by Powermarker version 3.25 and drawn using the software FigTree version 1.3.1 [35].

### Calculation of genome-specific relative kinship

Using the software package SPAGeDi [36], a selection of 8,000 SNPs, from the 24,994 with MAF ≥0.5, was used to calculate the relative kinship within the A- and C-subgenomes. For this purpose, 4,000 SNPs were randomly selected from *B. napus* A-subgenome chromosomes and 4000 from C-subgenome chromosomes. Negative values between two individuals, indicating that there was less relationship than that expected between two random individuals, were corrected to 0 as suggested by Yu *et al*. [37].

### Analysis of linkage disequilibrium

To investigate chromosome-wide and genome-specific patterns of linkage disequilibrium, the software package TASSEL 4.0 [38] was used to estimate LD (*r*^2^) on each chromosome and across the A- and C-subgenomes, respectively, using the 24,994 unique SNPs with MAF >0.5 and set a cut-off value of *r*^2^ = 0.1 to compare the extent of LD decay. We combined marker pairs into distance intervals, rather than considering them individually, to reduce the influence of outliers and to obtain a better visual description of the LD decay with distance. The genetic intervals of 44 regions were used in this study. As described by Yan *et al*. [17], the *r*^*2*^ value for a marker distance of 0 Kb was assumed to be 1.

### Haplotype block structure

HAPLOVIEW v4.2 [39] was used to estimate haplotype block structure in the 203 Chinese semi-winter rapeseed accessions across the 24,994 unique SNPs. The method followed for block definition was previously described by Gabriel *et al*. [40], who defined ‘strong LD’ if the one-sided upper 95% confidence bound of D’ is higher than 0.98 and if the lower bound is above 0.70.

### Haplotype block structure of homoeologous QTL regions

The genomic positions of known QTL for seed glucosinolate content on chromosome A02/C02 and A09/C09, and for erucic acid content on chromosomes A08/C03 (mapped in detail by Delourme *et al*. [26]) were localised by a BioEdit local BLAST search [41] using SNPs spanning the QTL confidence intervals.

A chromosome-scale alignment of the selected seed quality QTL regions was subsequently performed using the large-scale genome synteny tool SyMAP version 4.2 [42] (Additional file 4; Figure 7).

The genomic sequences of the overlapping homoeologous QTL regions for the two seed quality traits were used to search in the *A. thaliana* database (http://www.arabidopsis.org/Blast/) for genes annotated to seed glucosinolate, fatty acid or oil biosynthesis (Additional file 5). The haplotype block structure within the homoeologous QTL regions was studied in detail using HAPLOVIEW v4.2 to describe local LD around trait-relevant genes within in these regions. A heatmap comparing the LD structure across the QTL regions in the respective homoeologous A- and C-subgenome chromosomes were drawn using the R package LDheatmap [43].

## Electronic supplementary material

Additional file 1: **Genotype matrix, flanking sequences and genomic positions (best BLAST hit against the Darmor-** ***Bzh B. napus*** **V4.1 reference genome) for the subset of 24,994 high-quality, polymorphic, single-locus** ***Brassica napus*** **SNP markers with MAF ≥0.05, as used for the LD analyses.** SNP allele calls were generated in 203 *B. napus* breeding lines using the Illumina 60kSNP Infinium Brassica Consortium Array (Illumina Inc., San Diego, USA. (XLSX 20 MB)

Additional file 2: **Proportional memberships in subpopulations as defined by Structure.** (XLSX 32 KB)

Additional file 3: **Integrated map showing genomic positions of SSR and SNP marker sequences from QTL for seed glucosinolate (GLS) and erucic acid content, identified by BLAST searches onto A- and C-subgenome chromosomes from the** ***Brassica napus*** **Darmor-Bzh** **reference genome.** (XLSX 54 KB)

Additional file 4: **Details of synteny alignments for QTL positions for seed glucosinolate (chromosomes A02 and A09) and erucic acid (A08) into homoeologous regions of chromosomes C02, C09 and C03, respectively.** (XLSX 32 KB)

Additional file 5: **Detailed information on putative functional candidate genes and LD (haplotype block) analysis within the investigated QTL intervals for seed glucosinolate (GLS) and erucic acid content.** (XLSX 19 KB)

Additional file 6: **Comparative analysis of the extent of LD across homologous QTL for a,b) seed glucosinolate content (GLS) on homoeologous chromosomes a) A02/C02 and b) A09/C09, and c) erucic acid content on homoeologous chromosomes A08/C03.** The colored plots represent the pairwise LD across the respective homoeologous QTL regions, while the framed triangles represent regions with strongly conserved LD (LD blocks). The red and black small solid triangles represent positions of putative functional candidate genes, corresponding to Figure 8. (PDF 481 KB)
