# Supplementary figures and images for: Sub-genomic selection patterns as a signature of breeding in the allopolyploid Brassica napus genome
Source: BMC Genomics. 2014 Dec 23;15(1):1170. doi: 10.1186/1471-2164-15-1170 (PMC4367848; doi:10.1186/1471-2164-15-1170)

**a**

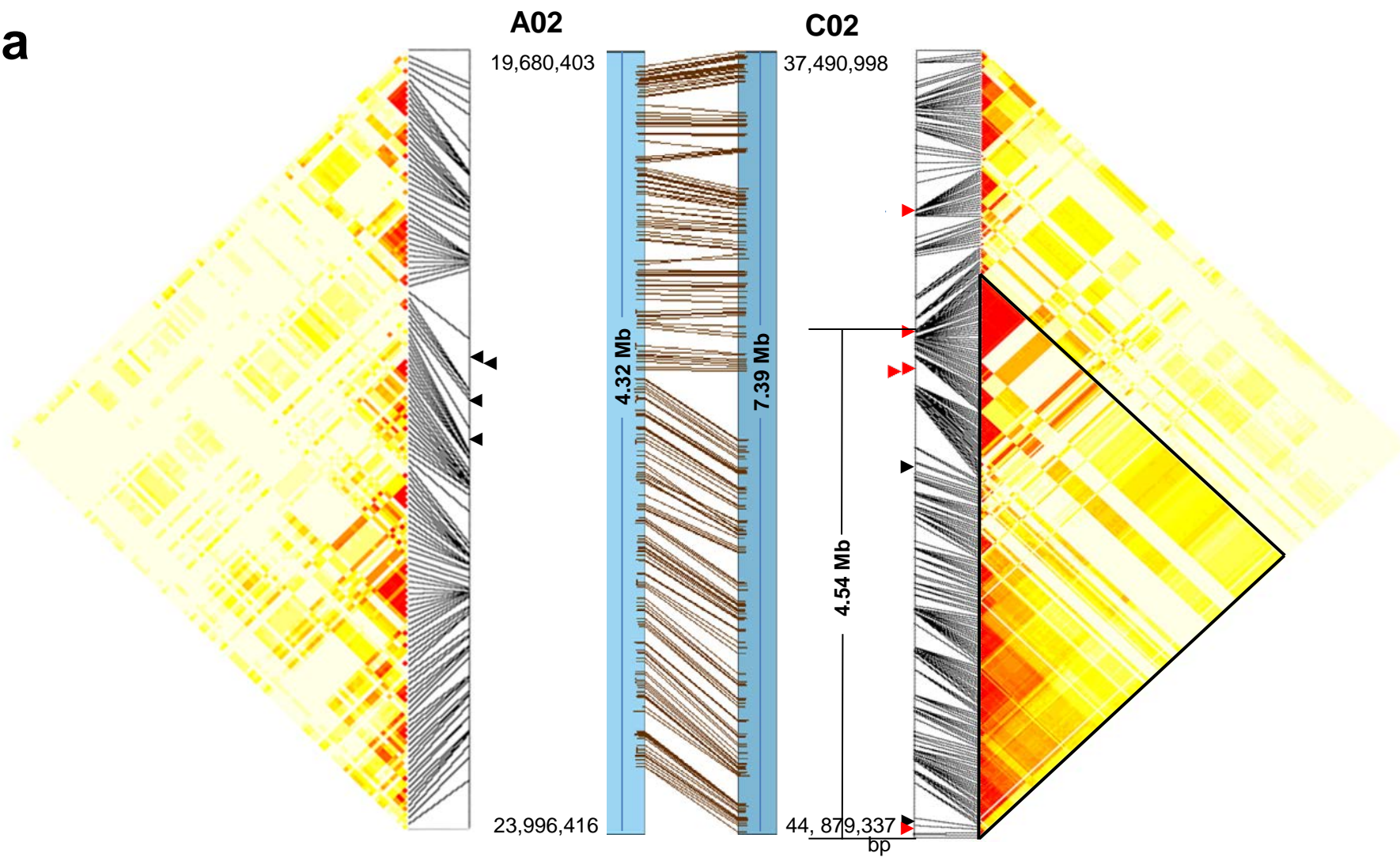

**b**

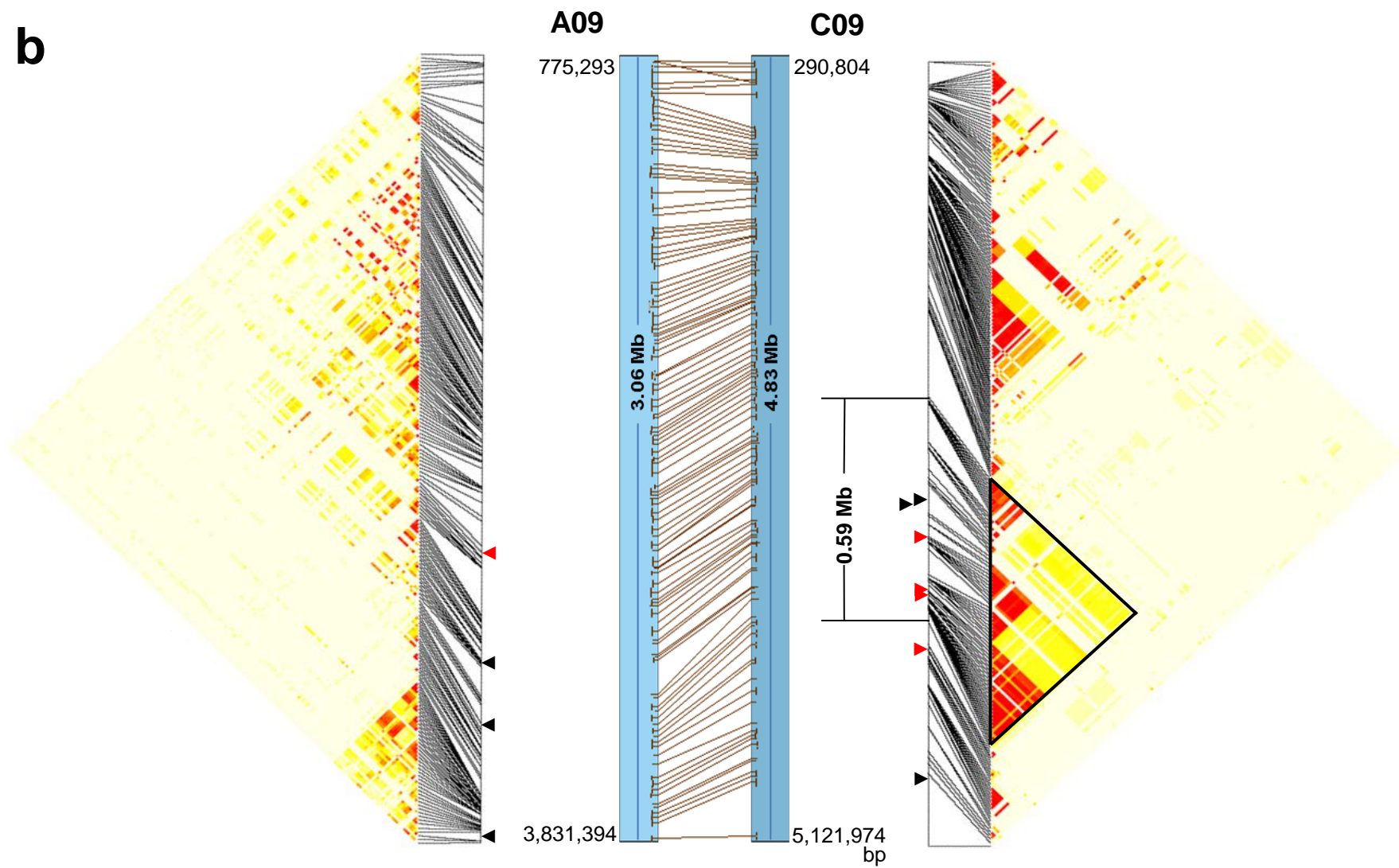

**C**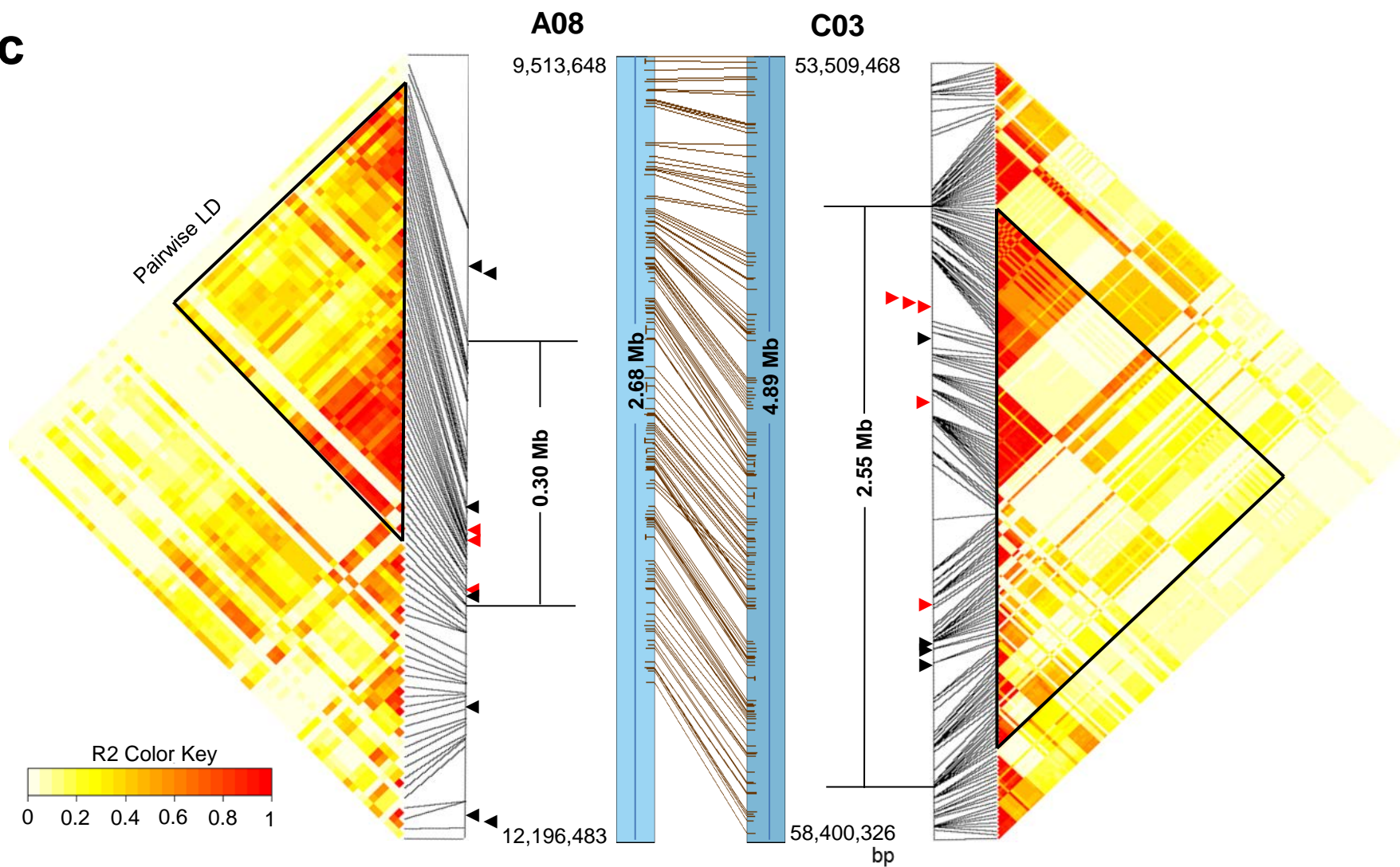

Supplement: Supplementary file 6 — Additional file 6: Comparative analysis of the extent of LD across homologous QTL for a,b) seed glucosinolate content (GLS) on homoeologous chromosomes a) A02/C02 and b) A09/C09, and c) erucic acid content on homoeologous chromosomes A08/C03. The colored plots represent the pairwise LD across the respective homoeologous QTL regions, while the framed triangles represent regions with strongly conserved LD (LD blocks). The red and black small solid triangles represent positions of putative functional candidate genes, corresponding to Figure 8. (PDF 481 KB) [file 12864_2014_6914_MOESM6_ESM.pdf]
